# When and How to Interpret Null Results in NIBS: A Taxonomy Based on Prior Expectations and Experimental Design

**DOI:** 10.3389/fnins.2018.00915

**Published:** 2018-12-11

**Authors:** Tom A. de Graaf, Alexander T. Sack

**Affiliations:** ^1^Section Brain Stimulation and Cognition, Department of Cognitive Neuroscience, Faculty of Psychology and Neuroscience, Maastricht University, Maastricht, Netherlands; ^2^Maastricht Brain Imaging Centre, Maastricht, Netherlands

**Keywords:** TMS, TES, bayes, localization, null, negative, inference

## Abstract

Experiments often challenge the null hypothesis that an intervention, for instance application of non-invasive brain stimulation (NIBS), has no effect on an outcome measure. In conventional statistics, a positive result rejects that hypothesis, but a null result is meaningless. Informally, however, researchers often do find null results meaningful to a greater or lesser extent. We present a model to guide interpretation of null results in NIBS research. Along a “gradient of surprise,” from Replication nulls through Exploration nulls to Hypothesized nulls, null results can be less or more surprising *in the context of prior expectations, research, and theory*. This influences to what extent we should credit a null result in this greater context. Orthogonal to this, experimental design choices create a “gradient of interpretability,” along which null results of an experiment, *considered in isolation*, become more informative. This is determined by target localization procedure, neural efficacy checks, and power and effect size evaluations. Along the latter gradient, we concretely propose three “levels of null evidence.” With caveats, these proposed levels C, B, and A, classify how informative an empirical null result is along concrete criteria. Lastly, to further inform, and help formalize, the inferences drawn from null results, Bayesian statistics can be employed. We discuss how this increasingly common alternative to traditional frequentist inference *does* allow quantification of the support for the null hypothesis, relative to support for the alternative hypothesis. It is our hope that these considerations can contribute to the ongoing effort to disseminate null findings alongside positive results to promote transparency and reduce publication bias.

## Introduction

With advent of digital-only journals, attention the to the downsides of publication bias, and preregistration of experiments, the call for dissemination of null results becomes louder. And indeed, it appears that null results are more often and more easily accepted for publication (see current issue). We support this development not only because we believe the community should have a more complete view of performed experiments (pragmatic argument), but also because we believe null results can be meaningful (interpretability argument). Seven years ago (de Graaf and Sack, 2011) we argued against a dichotomous distinction of positive and negative findings in non-invasive brain stimulation (NIBS) research, discussing criteria that could raise the interpretability of null results. We opened our paper with the familiar adage: absence of evidence is not evidence of absence. We then spent the remainder of the article arguing against it.

Of course, the core message is sound; absence of evidence does not necessarily, or always, imply evidence of absence. In classical statistics, frequentist inference, null results are formally meaningless: there was insufficient evidence in the dataset to reject the null hypothesis, but that is all one can say. The *P*-value reflects only the likelihood of obtaining an effect minimally as large as observed in the sample (e.g., difference between condition means), on the assumption that the null hypothesis is true. The *P*-value does not translate into a likelihood that the alternative hypothesis is true or false, and does not reflect the likelihood that the null hypothesis is true (Wagenmakers, 2007; Wagenmakers et al., 2018b). From a formal statistical point of view, a null result thus never constitutes evidence of absence (i.e., evidence for the null hypothesis). But that fact primarily reflects the constraints and limitations of this dominant statistical framework. Informally, null results can be less or more convincing, less or more meaningful, to an experienced researcher. In this article, we analyze on what grounds such meaning is assigned.

Brain stimulation research methodology has developed rapidly in the last decade, with increasingly sophisticated paradigms and applications (Sandrini et al., 2010; Herrmann et al., 2015; Romei et al., 2016; Ten Oever et al., 2016). But many of those still share a fundamental aim of NIBS: to evaluate the causal role, or functional relevance, of a given brain region/mechanism. Such a brain process is often initially found to correlate to some cognitive, emotional, or behavioral function, in a neuroimaging experiment with fMRI or EEG for example, and is thus *possibly* of crucial importance. But it might also be epiphenomenal, perform a related but different function, or occur as a consequence of still other brain processes that co-occur and are in fact the actual substrate for the task at hand (de Graaf et al., 2012). In this light, NIBS offers something unique in neuroscience: the ability to bring brain activity or specific brain mechanisms (e.g., oscillations) in specific regions or even whole networks under transient experimental control, allowing assessment of their causal relevance (Sack, 2006; de Graaf and Sack, 2014).

But the question whether a brain process is causally relevant has two possible outcomes: either it is (positive finding), or it is not (null finding). Considered this way, an *a priori* rejection of any outcome that is not a positive finding, i.e., complete rejection of null results as not informative, means one can only accept one of those two outcomes. It means NIBS experiments are a waste of time and resources if the null hypothesis is in fact true. It means that confirming the hypothesis that a brain process is *not* functionally relevant is not possible, and thereby any experiment to test it is doomed from the start. It has traditionally also meant that NIBS experiments with null results were (more) difficult to publish, preventing transparency, and completeness in the available NIBS literature. In sum, this restrictive view severely limits the usefulness of, and range of experimental questions open to, NIBS research.

If we do not want to categorically reject NIBS null results, we need to reflect on what can make them meaningful. Certain design decisions and parameters particularly contribute to the interpretability of NIBS null results, including the localization procedure, implementation of neural efficacy checks, and power and effect size. So how can one optimize experimental design or planned data analysis approaches prior to an experiment to maximize the interpretability of potential null results? And after obtaining null results, or reading about null results in publications, how can we assess how informative they are? How much do we let them change our beliefs and inform our own work? In this article, we discuss these issues with the still dominant frequentist inference framework in mind. But we also discuss an alternative statistical framework, Bayesian inference, which is not subject to the same formal limitations. We first outline factors that make NIBS null results particularly difficult to interpret, and how to address them. We then present conceptual handholds to evaluate null results along two orthogonal gradients, leading to a classification scheme of “levels of null evidence.” Lastly, we will explain what Bayesian analysis can contribute to such evaluation in a more formal and quantitative fashion.

## What Makes Null Results Difficult to Interpret in NIBS?

In de Graaf and Sack (), we discussed a perceived “dichotomy of meaningfulness” in transcranial magnetic stimulation (TMS) research: positive results were considered meaningful, negative results were considered meaningless. In part this might be attributable to the constraints of frequentist inference, but we suggested there were additional concrete arguments against null result interpretation specifically in NIBS: the localization argument, the neural efficacy argument, and the power argument. Though then focused on TMS, these arguments largely apply when it comes to other forms of NIBS, such as low-intensity transcranial electrical stimulation (TES).

According to the *localization argument*, one cannot be sure that the correct anatomical, or more importantly functional, area was stimulated with NIBS. Many NIBS studies still base their target localization on Talairach coordinates, MRI landmarks, or even skull landmarks. This can certainly be appropriate, scientifically and/or practically, but it means that in many participants the “functional hotspot” underlying a task/behavior might not be affected by NIBS. With TMS, error additionally contributes, with shifting or tilting coils, moving participants, or human error in initial coil placement. With TES, selecting electrode montages is not trivial. Even if a large electrode is placed on the skull, almost certainly covering an underlying functional hotspot, the exact individual anatomy as well as reference electrode placement may determine which neurons are most affected and how effectively they are modulated (Miranda et al., 2006; Wagner et al., 2007; Bikson et al., 2010; Moliadze et al., 2010).

The *neural efficacy argument* appears related but reflects a separate concern. While “localization” refers to the success of cortical targeting, the “neural efficacy” argument reflects uncertainty about whether there was any effective stimulation at all. In each participant, or even the whole sample: did the NIBS actually modulate neural activity? In TMS, the infinite parameter space (number of pulses, pulse shape, pulse width, intensity, (nested) frequency, coil geometry, etc.) allows for a nearly infinite number of protocols (Robertson et al., 2003), many of which might not achieve neural stimulation. Moreover, between participants anatomy differs, in terms of gyrification or distances between coil and cortex (Stokes et al., 2005). The neural efficacy argument thrives in TES methodological discussions as well, with ongoing investigation on how much of the electrical current even reaches the cortex (Vöröslakos et al., 2018). In short, in NIBS one can often doubt in how many participants neural stimulation/modulation was successful, aside from which cortical area was targeted.

The *power argument* is more general but applies to NIBS research also. Perhaps a positive finding was not obtained, simply because the experimental design lacked statistical power. Aside from the usual sources of noise in experimental data, the methodological uncertainties inherent to NIBS research, of which localization and neural efficacy are examples, only exacerbate this concern. Moreover, it appears that inter-individual differences in response to NIBS are substantial (Maeda et al., 2000). For instance, inter-individual differences in network states (Rizk et al., 2013), structural organization of the corpus callosum (Chechlacz et al., 2015), and neuronal oscillatory parameters (Goldsworthy et al., 2012; Schilberg et al., 2018) have been related to NIBS response. If unknown, or not taken into account, such differences can contribute strongly to reduced statistical power on the group level. The power argument leaves one with the uncomfortable question: perhaps more trials per condition, or more participants in the sample, or even a small change in experimental tasks or design, could have yielded a positive result after all. So how meaningful is a null result in NIBS research, really?

To summarize, if no effect was found: (1) perhaps the intended cortical region was not successfully targeted, (2) perhaps no neural stimulation/modulation took place in some or all participants, or (3) perhaps the experiment lacked power. These arguments indeed make it difficult to draw strong conclusions from null results in NIBS research. But in our view they do not make NIBS null results categorically uninformative (“dichotomy of meaningfulness”). Above, we argued why such a dichotomy would be unfortunate and even wasteful, given the original mission of NIBS to determine whether a brain process is, *or is not*, functionally relevant for a task. Below, we discuss what can be done in terms of experimental design to address these three arguments, and in turn how one can evaluate such design choices in one’s own work, or research published by others, to evaluate how informative a null result is.

## What Can Make Null Results Easier to Interpret in NIBS?

We suggest that the null results of any NIBS experiment, considered in isolation, can be interpreted along a “gradient of interpretability.” Where null results fall along this gradient, i.e., how strongly we allow ourselves to interpret them, is determined by the extent to which the localization argument, neural efficacy argument, and power argument can be countered through experimental design decisions and analysis.

One way to combat the localization argument is through hardware selection. Figure-8 TMS coils stimulate more focally than some (older model) circular coils (Hallett, 2007), electrical currents are more concentrated under the central electrode in a high-density TES montage compared to conventional large electrodes (Edwards et al., 2013). But focality does not help if it is aimed at the wrong cortical target. The localization argument against interpreting null results becomes *less* problematic as the *localization procedure* becomes more sophisticated. We previously compared the statistical power inherent to different TMS target site localization procedures, including anatomical landmarks, Talairach coordinates, individual MRI-based landmarks, and individual fMRI-based functional localizers, using frameless stereotactic Neuronavigation to determine and maintain coil positioning (Sack et al., 2009). The number of participants required to obtain a statistically significant effect (calculated based on obtained effect size for each method) rose rapidly, from only 5 using individual functional localizer scans, to 47 using the anatomical landmark (EEG 10–20 P4 location) approach. As power rises with localization procedure sophistication, for a given sample size the interpretability of a potential null result rises also (although see e.g., Wagenmakers, 2007; Wagenmakers et al., 2015). For TES, exciting developments in computational modeling of current flows, in increasingly realistic head models, similarly decrease the strength of the localization argument. Such modeling provides insight about how strongly, and where, electrical currents affect underlying neuronal tissue (e.g., Datta et al., 2009).

Clearly such modeling in TES also has a bearing on the neural efficacy argument: modeling increases confidence that current was sufficiently strong to reach and affect the cortical target. This is not to say that all problems are solved for TES, because firstly modeling is not trivial, and secondly a model of current density across cortex does not yet reveal what the neural effects are of this current density. Similarly for TMS, unless it is combined with simultaneous imaging (fMRI, EEG), what happens in the brain after each pulse is principally unknown to us. Maybe the protocol is insufficiently strong to induce action potentials, maybe the induced action potentials are insufficient to induce cognitive/behavioral effects, maybe the stimulation does affect the targeted area but other areas in a network compensate for the insult (Sack et al., 2005). To combat at least some of these concerns, one might implement what we previously called a *neural efficacy check* (de Graaf and Sack, 2011).

One way to see if NIBS affected brain activity, is to indeed actually measure brain activity, by combining NIBS with fMRI or EEG (Reithler et al., 2011), or all together simultaneously (Peters et al., 2013): the concurrent neuroimaging approach. Specifically for TMS, if the target region has a behavioral marker (motor response, phosphene perception), both the localization and neural effects of a TMS protocol can be verified using such markers independently from the behavioral tasks of interest. Hunting procedures, using independent tasks *known* to be affected by certain TMS protocols over the target regions, can also be used to achieve the same thing (Oliver et al., 2009). Ideally, however, the experimental design includes not only the main experimental condition of interest, on which a positive or null result can be obtained, but also a second experimental condition on which a positive result is expected. For example, we applied offline rTMS to frontal cortex in a bistable perception paradigm with a passive viewing condition and a voluntary control condition using the same stimuli (de Graaf et al., 2011b): two experimental conditions with their own controls. TMS modulated bistable perception in the voluntary control condition (positive result) but not in the passive viewing condition (null result). The presence of the positive finding made the null result for passive bistable viewing more meaningful to us, since it inspired confidence that our TMS protocol and procedures indeed had neural effects with the potential to affect behavior.

In that study, passive bistable viewing behavior was very much unchanged. It was not a case of a small effect in the hypothesized direction not reaching significance. And this is really all one can do against the power argument: ensure *sufficient power* in the experimental design through *a priori* sample size calculations, and *evaluate the effect size* after data collection. The possibility that an even larger sample size, and more reliable estimation through more trials, could eventually lead to a statistically significant positive finding, is irrefutable. In fact, it is inevitable: in classical frequentist hypothesis tests, with large enough sample sizes even true null hypotheses will be rejected (Wagenmakers, 2007). This is not unique to NIBS research. What may be to some extent unique to NIBS research, and indeed an exciting development, is the inclusion of individual markers that predict response to NIBS. As we continue to discover sources of inter-individual variability, we can either select participants (Drysdale et al., 2017), adapt protocols (Goldsworthy et al., 2012), or refine our statistical analyses to increase detection power.

## A Gradient of Surprise and a Gradient of Interpretability

**FIGURE 1:**
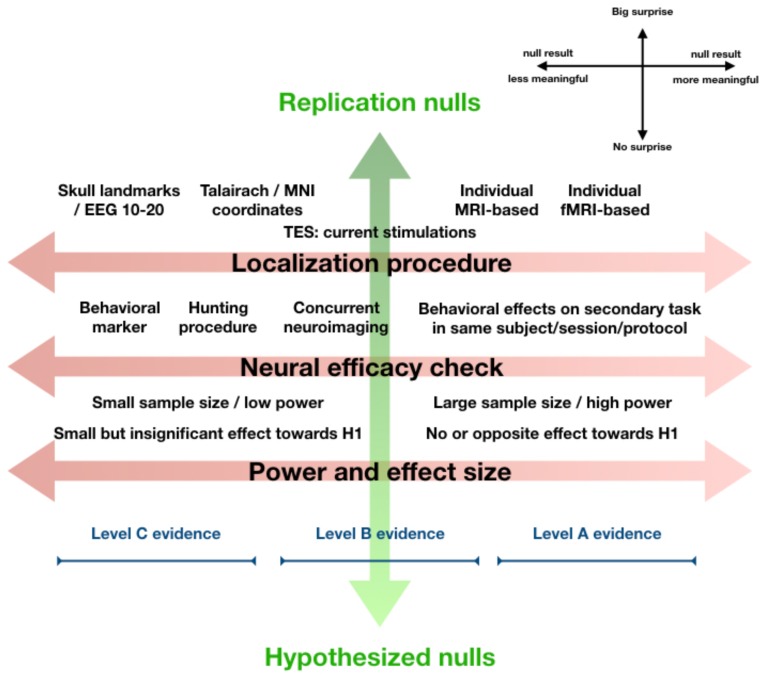
Orthogonal gradients of surprise and interpretability. A null result in an experiment aiming to replicate a well-established finding is very surprising. A null result that was hypothesized is not at all surprising. A null result in an exploratory study with no prior expectations can be received neutrally (middle of the continuum, not shown). The level of surprise (vertical axis) essentially reflects how a null finding relates to our prior expectations. From a Bayesian perspective, even without Bayesian statistics, we should let this level of surprise (if justified based on theory or previous research) influence to what extent we let the result “change our beliefs.” This explicitly refers to interpretation of a null result in the greater context of knowledge, theory, and prior research. Orthogonal to this, one might evaluate the experiment and its parameters in terms of localization procedure, neural efficacy checks, and power and effect size, together making up a gradient of interpretability (horizontal axis). This continuum reflects how informative we should consider the null result *in isolation*, ignoring expectations, theory, or previous research. The figure displays our view on how design choices impact the interpretability of a null finding along this dimension (toward the right is more informative, see legend top-right). At the bottom, we schematically visualize how the collective of such “leftward” design choices can place a null result into the “Level C evidence category,” which means the null result in isolation is not very informative, while the collective of such “rightward” design choices can place a null result in the highest “Level A evidence” category, which means a null result appears informative and should be taken seriously. A few caveats are important. This figure aims to visualize concepts discussed in more detail in main text, and how they relate to each other. The visualization of Level C through Level A evidence is meant to make intuitive how they roughly fit into this overview, our proposal for what exactly differentiates Level A through C evidence is in main text. Lastly, we do not suggest that every design decision “on the right” in this figure is always best for every experiment, or that experiments yielding Level C evidence are somehow inferior. Note also that the figure reflects how design factors influence how informative *null findings* are, it does *not* apply to positive findings in a straightforward way.

In Figure 1, we distill the discussion so far into a conceptual model to help us evaluate null results in NIBS. The model contains two orthogonal axes. Horizontally, the relevant parameters and design decisions in an experiment, discussed above, are mapped along the “gradient of interpretability.” Where an experiment ranks along this dimension determines how informative its null result is, *considered in isolation*. Vertically, the “gradient of surprise” indicates how unexpected the null result is, *in the context of prior research and theory*.

Along the *gradient of surprise*, we find Replication nulls on one end, Exploration nulls in the middle, and Hypothesized nulls at the opposite end. Sometimes a positive finding is strongly expected, based on previous research or strong theoretical background, but not found. For instance, a null result in an experiment explicitly designed to replicate a well-established finding seems most surprising (Replication null). On the other hand, some null results can be received more neutrally, if the research was more exploratory and a result could “go either way.” For instance, because research is very original and breaks new ground in uncharted territory, or because competing theories with approximately equal support make opposite predictions for the outcome of an experiment. Thus, Exploration nulls sit in the middle of the continuum. At the other end of the gradient are Hypothesized nulls. These may be something of a rarity still, since not many experiments are explicitly designed to obtain null results (in the experimental condition). Perhaps this is because null results seem less interesting or impactful, or simply because negative findings are *a priori* considered meaningless for reasons discussed above. Either way, a null result that was expected is of course least surprising.

Along the gradient of interpretability, multiple factors contribute to null result interpretability. Among these the selected localization procedure, existence of neural efficacy checks, and the power and effect size. Together, these “design choices” help determine to what extent a null result can be interpreted. But these are several factors that contribute to null result interpretability along this same dimension somewhat independently, and additively. Combining this with the breadth of approaches, techniques, parameters, and even further design decisions in NIBS research, it seems the landscape of null results is rather complex. Therefore, we propose three concrete “Levels of evidence” for NIBS null results (Levels A, B, and C), as heuristic guidelines to help us determine how informative the null results from an individual experiment are. These Levels of evidence explicitly apply to the gradient of interpretability. They are visually added to Figure 1, but only to make intuitive how they are ordered along this continuum.

## Three Levels of Null Evidence

Our heuristic taxonomy of null result interpretability demarcates three levels of null evidence, from least (Level C) to most interpretable (Level A) null results. We want this taxonomy to be useful independent of Bayesian analysis, but will later comment on how Bayesian analysis fits into this scheme.

*Level C evidence* is assigned to null results from experiments with localization procedures not based on individual anatomy or functional mapping (i.e., landmark- or Talairach-/MNI-coordinate based) and no neural efficacy checks. Often, such null results may result from exploratory studies, studies with exploratory NIBS parameters, high risk studies, small-scale student projects, etc. Such studies might be meaningful to share with the community, for the sake of transparency. They could help others remain aware of what has been tried, which parameters, tasks, cortical sites, have been targeted under which procedures, even if the null result itself cannot be strongly interpreted (the Pragmatic argument; de Graaf and Sack, 2011). Opinions may differ, however, on what minimal level of sophistication an experiment requires for the ‘attempt’ to be meaningful to others and therefore published (see also de Graaf et al., 2018).

*Level B evidence* is assigned to null results from experiments with *either* sophisticated localization procedures (individual MRI- or fMRI guided neuronavigation for TMS, current simulations in individual anatomy for TES) *or* neural efficacy checks (behavioral markers, hunting procedure, concurrent neuroimaging). Thus, either through individual neuronavigation/modeling, or through a neural efficacy check, one is relatively *sure that the intended cortical region was targeted*. For level B evidence, it is not necessary to have the strongest form of a neural efficacy check, demonstrating behavioral effects on a related task by the NIBS protocol used also in the condition leading to the null result. In the absence of Bayesian analysis: there should be clear absence of effects in the data despite – as far as could be determined *a priori* – sufficient statistical power for the selected sample size. Null results with Level B evidence should, in our view, always be acceptable for publication, irrespectively of whether the null result is surprising, exploratory, or hypothesized (not publishing just because of surprise contributes to publication bias).

*Level A evidence* is assigned to null results with individually calibrated localization procedures (same as in level B) *and* strong neural efficacy checks. The neural efficacy checks should confirm not only successful targeting of the intended cortical site, but successful neural stimulation/modulation, either by demonstrating behavioral effects in an experimental condition with related stimuli/task and identical NIBS protocol, *or* by revealing neural effects of the NIBS intervention in appropriate regions/networks/mechanisms through concurrent neuroimaging. Level A classification further requires clear absence of effects in the experimental condition, despite sufficient statistical power for the given sample size. Level A null results constitute sufficiently meaningful contributions to warrant dissemination.

Note that these descriptions of Levels C through A reflect a first proposal, and the criteria are open to debate. It is an unavoidably flexible classification scheme, since multiple factors along the same continuum (see Figure 1) contribute to null result interpretability. Moreover, with continuing developments, certain design choices and procedures may fall between such classifications. For instance, is the cortex-based alignment approach for TMS localization, in which group fMRI-based Talairach coordinates can reliably be mapped onto individual MRI-based brain anatomy, a Level B localization procedure or not (Duecker et al., 2014)? We therefore consider these Levels of evidence a rough classification that *may* be applied directly, but in equal measure can guide a more informal evaluation of null result meaningfulness.

## The Orthogonal Contributions of Interpretability and Surprise

We here discuss more explicitly how this model can guide informal assessment of null results. As mentioned, the gradient of interpretability underlying the Levels of evidence is orthogonal to the gradient of surprise, since it focuses on how informative an individual null finding is *in isolation*, ignoring our prior expectations or level of surprise by the outcome. But of course, the *a priori* likelihood of a positive finding or a null result, in other words our level of surprise at the obtained null result, *should* influence how we interpret and credit such a result in the *greater context* of previous research and theory.

In fact, this lies at the heart of the Bayesian school of thought: we integrate our prior beliefs with the new data, to come to a new belief. We suggest that evaluating a null result along the two gradients of our model can essentially guide an “informal Bayesian analysis.” In the informal procedure of integrating our prior understanding with the new data, we can for instance allocate less or more *weight* to the new data (null result) and less or more weight to our prior expectations. The weight allocated to the new data is determined by the position of the null result along the gradient of interpretability (horizontal in Figure 1), while the weight of the prior expectations directly relates to the level of surprise instigated by the null result (vertical in Figure 1).

For example, if a Level C null result is very surprising, because a dominant theory and several previous studies predicted a positive finding, we should not be so quick to reject the theory, and seriously consider the possibility that we obtained a Type-II error. And, of course, we should design a new experiment that would yield more informative null results. In contrast, a Level A null result could make us rethink the theory, or inspire follow-up experiments to determine what caused the discrepancy. Yet, in this scenario we still do not outright accept even a Level A null result. But had the very same Level A null result been obtained in the absence of prior expectations, because the experiment addressed a fundamentally new research question, then this Level A Exploration null result could guide our beliefs more strongly, even forming the starting point for a new theory.

These arguments are quite abstract, so perhaps it is useful to consider two more concrete examples. Imagine an experiment that fails to replicate a well-established TMS effect, suppression of visual stimuli by a single occipital TMS pulse at 100 ms after stimulus onset (Amassian et al., 1989; de Graaf et al., 2011a,b,c, 2015). This Replication null would very much surprise us, given the extensive literature supporting this effect (de Graaf et al., 2014). *Independently* of this surprise, we would consider the null result less meaningful if the TMS coil was simply positioned just a few centimeters above the inion, versus if the TMS coil elicited phosphenes in the retinotopic stimulus location (neural efficacy check based on perceptual marker), or were neuronavigated to an individual hotspot in V2 corresponding to the retinotopic stimulus location (sophisticated localization procedure). Similarly, if an imaginary fMRI experiment found that appreciation of magic tricks scales with posterior parietal cortical BOLD activation, one might follow it up with an exploratory TMS experiment. Not finding any reduced appreciation for magic tricks after inhibitory TMS, an Exploration null result, might not necessarily surprise us. But, again *independently* of our lack of surprise, we would find the null result much more informative if we had neuronavigated the TMS coil to individual hotspots as compared to placing the TMS coil over the EEG 10-20 P4 location (sophisticated localization procedure), or if the reduction in magic appreciation was zero as compared to a reduction in magic appreciation that was in the right direction but just failed to reach significance (effect size evaluation).

## The Bayesian Inference Approach

In sum, positioning a null result along the gradients of interpretability and surprise can help us in the interpretation of such a null result and the extent to which we should let it change our beliefs. We even called this an “informal Bayesian analysis.” But, as mentioned previously, *formally* null results cannot be interpreted at all. At least, not in the conventional frequentist statistical framework of classical null hypothesis testing yielding *P*-values. Some examples of limitations of *P*-values (Wagenmakers, 2007; Wagenmakers et al., 2018b):

1. we cannot interpret any outcome as supporting the null hypothesis;
2. many find it difficult to correctly interpret *P*-values (Nickerson, 2000; Verdam et al., 2014);
3. hypothesis tests are biased against the null hypothesis, since the *P*-value ignores both predictions of and *a priori* likelihood of the alternative hypothesis, only testing predictions of the null hypothesis;
4. *P*-values not only fail to quantify evidence for the null hypothesis, they also fail to quantify evidence for any alternative hypothesis;
5. the entire analysis framework relies on imaginary replications that form a sampling distribution, required to obtain a *P*-value.

Perhaps due to such limitations, *P*-values are increasingly reported alongside additional statistics, such as effect sizes and confidence intervals. But they remain the mainstay of inference in NIBS research. Classical null hypothesis testing does not consider the prior likelihood of null or alternative hypotheses. It does not account for how likely any outcome is based on previous research, solid theoretical foundations, or just common sense. As a result, the same *P*-value coming out of the same hypothesis test might convince an experienced researcher in one case, but not at all in another case. This applies to positive findings as well as negative findings. As such, experienced researchers do not merely take *P*-values at face value, but generally evaluate them in greater context already.

The Bayesian approach is fundamentally different, and its formal implementation as an alternative statistical approach is becoming increasingly widespread. It can incorporate those prior beliefs we mentioned, formalizing in the Bayesian analysis framework some of what experienced researchers informally do when dealing with frequentist analysis outcomes. Model priors reflect the *a priori* likelihood of null or alternative hypotheses. But also prior expectations about particular parameters, such as an effect size in a population, can be quantified under the null and alternative hypothesis, prior to data collection. The analysis takes those priors, then takes the collected data from an experiment, and integrates these sources of information (updates the prior distribution) to yield a “posterior distribution.” The posterior distribution is our updated belief about the effect under investigation, yields a certain “credible interval” when it comes to an estimated parameter (a 95% credible interval for a parameter means we are 95% sure the parameter lies in that interval), and can directly be evaluated for hypothesis testing.

Bayesian hypothesis testing uses the priors and the available data to weigh the evidence for and against the null and alternative hypotheses. The analysis can yield a single numerical value called the *Bayes Factor*, which quantifies the relative evidence. It quantifies which hypothesis is more supported by the data and to what extent, or in other words, which of the two hypotheses best predicted the data and by how much. This approach, as any, has its own limitations; the quantification of priors may not always be trivial, for example, and just as the classical *P* < 0.05 criterion for classification of a significant effect is arbitrary, the Bayes Factor (BF) is descriptively interpreted as providing “anecdotal,” “moderate,” or “strong” evidence for either hypothesis at some arbitrary boundaries. However, the approach does offer benefits and capabilities not afforded by classical statistics. In the context of the current discussion, Bayesian analysis has one particularly important advantage: in Bayesian analysis, one *can* interpret a null result and formally draw conclusions based on it (see also in this issue: Biel and Friedrich, 2018).

**FIGURE 2:**
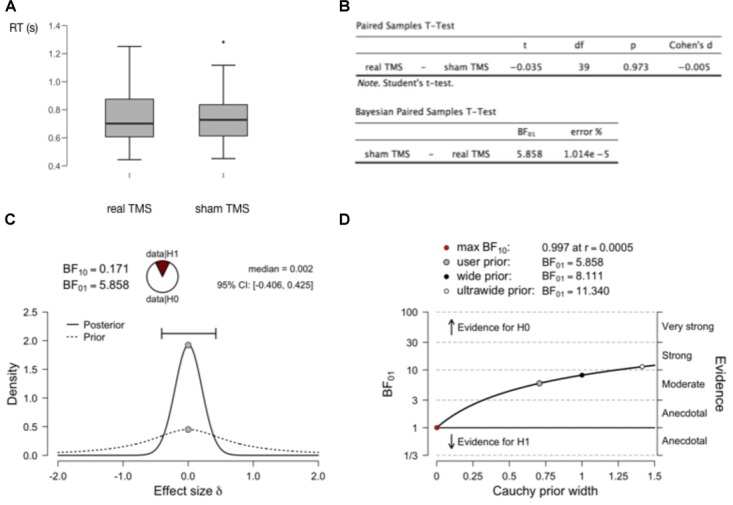
Bayesian analysis to assess support for a null hypothesis. **(A)** Results of two fictional within-subject conditions in a (random-walk) generated dataset with 40 virtual observers. There does not seem to be a meaningful difference in RT between both conditions. **(B)** A traditional paired-samples *t*-test provides no reason to reject the null hypothesis (*P* > 0.05). But it can provide no evidence to accept the null hypothesis. The Bayesian paired-samples *t*-test equivalent yields a BF01 = 5.858. This means that one is 5.858 times more likely to obtain the current data if the null hypothesis is true, than if the alternative hypothesis is true. In the recommended interpretational framework, this constitutes “moderate” evidence for the null hypothesis (BF > 3, but < 10 which would constitute “strong” evidence). **(C)** A plot of the prior distribution (dashed) and posterior distribution (solid), reflecting probability density (vertical axis) of effect sizes in the population (Cohen’s d, horizontal axis). In these tests, the prior distribution is conventionally centered around 0, but its width can be set by the user to reflect strength of prior expectations. The width of the posterior distribution reflects confidence about effect size based on the prior and the data: the horizontal bar outlines the “credible interval” which contains 95% of the posterior distribution density. This width/interval will be smaller with increasing sample size. The median of the posterior distribution (0.002) is a point estimate of the real effect size. BF10 here is simply the inverse of BF01, and the “wedge-chart” visualizes how much more likely one is to obtain the current data given H0 versus H1. **(D)** Since setting a prior (width) is not always straightforward, one can plot how much the outcome of the analysis (BF01, vertical axis) depends on the selected prior (horizontal axis). At the top are the BF values for a few points on the plot (user-selected prior, in this case the JASP-provided default of Cauchy prior width = 0.707, wide, and ultrawide prior). Clearly the evidence for the null hypothesis (likelihood of obtaining current data if null hypothesis is true) ranges for most reasonable priors from moderate to strong.

This article is not intended to be a (Bayesian) statistics tutorial, but it may be useful to provide an example with just a little bit more concrete information. In Figure 2 we show generated data from a simple fictional TMS experiment, measuring reaction times in a real TMS condition as compared to a sham TMS condition (2A). The figure shows the outcomes of a standard paired-samples *t*-test, as well at the outcomes of a Bayesian equivalent paired-samples test (2B). These analyses were all performed with the free and open-source statistical software package JASP (JASP Team, 2018; Wagenmakers et al., 2018a,b). A relevant outcome of the Bayesian test is the BF. The Bayes Factor BF10 reflects how much more likely the data were to occur under the alternative hypothesis as compared to the null hypothesis (1C). Bayes Factor BF01 is the inverse (1/BF10). If the BF is larger than 3, the evidence is considered “moderate,” if larger than 10, the evidence is considered “strong” (Wagenmakers et al., 2018a). If one is worried about the concept of priors, and how much of an influence a chosen prior has on the outcome of the analysis, this can simply be evaluated quantitively (2D). See the figure caption for further details.

## Bayesian Statistics and Levels of Evidence

Bayesian statistics can formally and quantitatively evaluate the strength of evidence of a null result. Since this is what we informally intended to achieve with a classification of Levels of null evidence, should we not simply demand Bayesian statistics instead? Or require certain ranges of BFs before we accept null results as Level B or A evidence? This is an interesting question, but currently we think not. Firstly, because Bayesian analysis is not yet commonplace, although it is increasingly implemented. This does not mean we doubt the approach, but we would like our model to be helpful also to researchers not ready to commit to Bayesian analysis. Secondly, the BF quantifies the support for the null (and alternative) hypothesis *in the data.* The gradient of interpretability, however, (also) ranks how much faith we should put in those data in the first place, based on certain experimental design decisions. As such, the results of Bayesian analysis (or classical inference) only become meaningful once we reach a certain level of confidence in the experimental design and its power to yield meaningful data.

Having said that, we do strongly advocate the application of Bayesian analysis, at least to complement classical hypothesis tests when presenting null results. We are hesitant to propose, for example, that a Level A classification can only be extended to a null result backed up by Bayesian statistics. We would, however, suggest that, to build a case against a null hypothesis based on a null result, adding Bayesian analysis is preferred and would usually make the case stronger. A Level A null result comes from an experiment with such a sophisticated design that its data should be considered meaningful. Therefore, formal Bayesian quantification of support for the null hypothesis would be meaningful also.

## Conclusion

This is an opinion paper, meant to provoke thought, insight, and discussion. Some of the ideas, and especially criteria for classification of null result interpretability, are somewhat arbitrary and may change with new insights, developments, or time. But the fundamental idea underlying it, is that not all null results are created equal. And it may be useful to reflect on what it is about null results, and especially the design of NIBS experiments underlying them, that allows us to consider them less or more meaningful. Our proposals here provide concrete heuristics, but are open to amendment, correction, or expansion.

## Author Contributions

All authors listed have made a substantial, direct and intellectual contribution to the work, and approved it for publication.

## Conflict of Interest Statement

The authors declare that the research was conducted in the absence of any commercial or financial relationships that could be construed as a potential conflict of interest.
